# Supplementary material for: Causal Relationship between Aging and Anorexia Nervosa: A White-Matter-Microstructure-Mediated Mendelian Randomization Analysis
Source: Biomedicines. 2024 Aug 16;12(8):1874. doi: 10.3390/biomedicines12081874 (PMC11351342; doi:10.3390/biomedicines12081874)
Supplement: Supplementary file 1 [file biomedicines-12-01874-s001.zip › Supplementary Figure/Figure S4/Singe SNP plot of MR analysis for TL to ubm-b-1508.pdf]

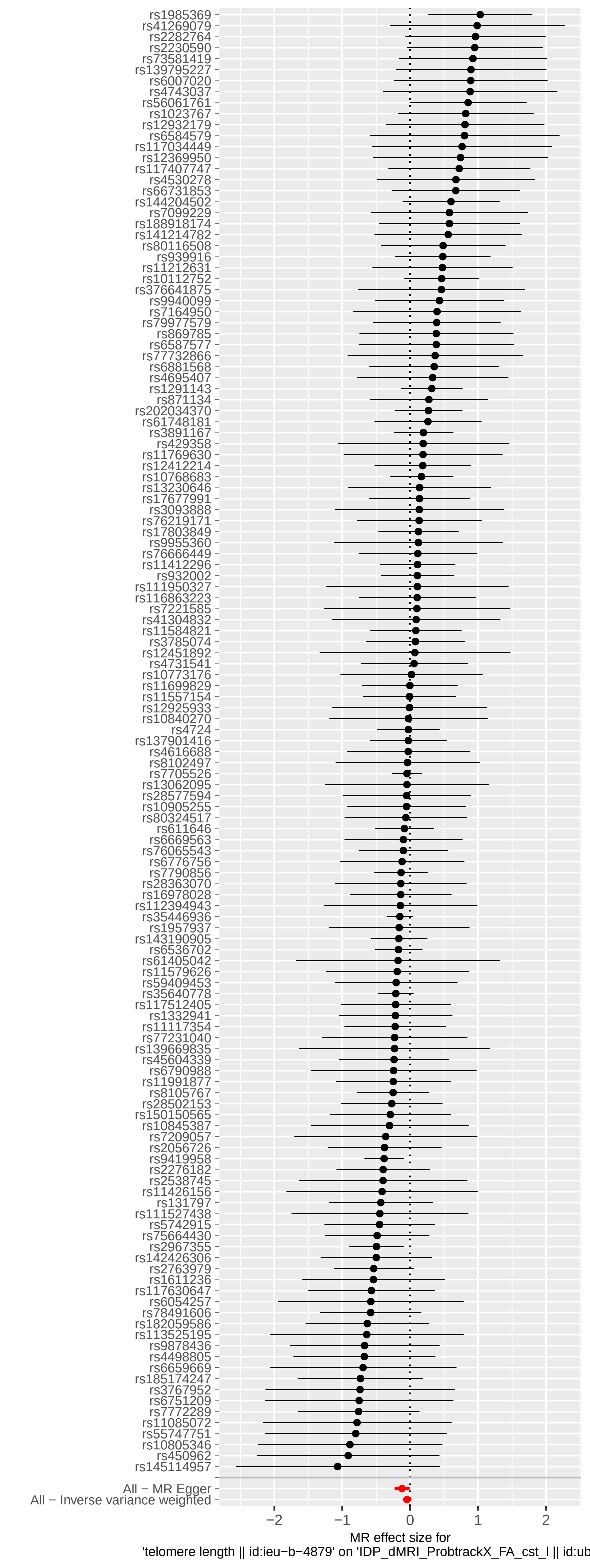
rs1985369
rs41269079
rs2282764
rs2230590
rs73581419
rs139795227
rs6007020
rs4743037
rs56061761
rs1023767
rs12932179
rs6584579
rs117034449
rs12369950
rs117407747
rs4530278
rs66731853
rs144204502
rs7099229
rs188918174
rs141214782
rs80116508
rs939916
rs11212631
rs10112752
rs376641875
rs9940099
rs7164950
rs79977579
rs869785
rs6587577
rs77732866
rs6881568
rs4695407
rs1291143
rs871134
rs202034370
rs61748181
rs3891167
rs429358
rs11769630
rs12412214
rs10768683
rs13230646
rs17677991
rs3093888
rs76219171
rs17803849
rs9955360
rs76666449
rs11412296
rs932002
rs111950327
rs116863223
rs7221585
rs41304832
rs11584821
rs3785074
rs12451892
rs4731541
rs10773176
rs11699829
rs11557154
rs12925933
rs10840270
rs4724
rs137901416
rs4616688
rs8102497
rs7705526
rs13062095
rs28577594
rs10905255
rs80324517
rs611646
rs6669563
rs76065543
rs6776756
rs7790856
rs28363070
rs16978028
rs112394943
rs35446936
rs1957937
rs143190905
rs6536702
rs61405042
rs11579626
rs59409453
rs35640778
rs117512405
rs1332941
rs11117354
rs77231040
rs139669835
rs45604339
rs6790988
rs11991877
rs8105767
rs28502153
rs150150565
rs10845387
rs7209057
rs2056726
rs9419958
rs2276182
rs2538745
rs11426156
rs131797
rs111527438
rs5742915
rs75664430
rs2967355
rs142426306
rs2763979
rs1611236
rs117630647
rs6054257
rs78491606
rs182059586
rs113525195
rs9878436
rs4498805
rs6659669
rs185174247
rs3767952
rs6751209
rs7772289
rs11085072
rs55747751
rs10805346
rs450962
rs145114957
All − MR Egger
All − Inverse variance weighted
−2
−1
0
1
2
MR effect size for
'telomere length || id:ieu−b−4879' on 'IDP_dMRI_ProbtrackX_FA_cst_l || id:ub
